# Distinct life cycle stages of an ectosymbiotic DPANN archaeon

**DOI:** 10.1093/ismejo/wrae076

**Published:** 2024-05-01

**Authors:** Vasil A Gaisin, Marleen van Wolferen, Sonja-Verena Albers, Martin Pilhofer

**Affiliations:** Department of Biology, Institute of Molecular Biology & Biophysics, Eidgenössische Technische Hochschule Zürich, Otto-Stern-Weg 5, 8093 Zürich, Switzerland; Molecular Biology of Archaea, Institute of Biology, University of Freiburg, Schänzlestr. 1, 79104 Freiburg, Germany; Molecular Biology of Archaea, Institute of Biology, University of Freiburg, Schänzlestr. 1, 79104 Freiburg, Germany; Department of Biology, Institute of Molecular Biology & Biophysics, Eidgenössische Technische Hochschule Zürich, Otto-Stern-Weg 5, 8093 Zürich, Switzerland

**Keywords:** Nanoarchaeota, archaeal symbiosis, contact-dependent interactions, extracellular appendages, archaeal cell biology

## Abstract

DPANN archaea are a diverse group of microorganisms that are thought to rely on an ectosymbiotic lifestyle; however, the cell biology of these cell–cell interactions remains largely unknown. We applied live-cell imaging and cryo-electron tomography to the DPANN archaeon *Nanobdella aerobiophila* and its host, revealing two distinct life cycle stages. Free cells possess archaella and are motile. Ectobiotic cells are intimately linked with the host through an elaborate attachment organelle. Our data suggest that free cells may actively seek a new host, while the ectobiotic state is adapted to mediate intricate interaction with the host.

## Graphical Abstract

**Graphical Abstract:**
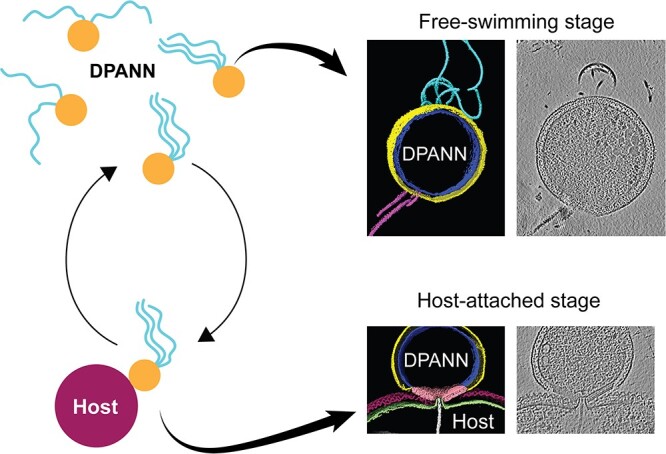

## Introduction

One advantage of a close symbiotic microbial lifestyle can be the possibility to access substrate that is bound in other living cells. The first ectosymbiotic relationship between two archaeal species was reported for *Nanoarchaeum equitans* and its host [1]. This archaeon is a member of the phylogenetically highly diverse group of DPANN archaea. DPANN is an acronym based on the lineages Diapherotrites, Parvarchaeota, Aenigmarchaeota, Nanohaloarchaeota, and Nanoarchaeota, which, among others, constitute this phylogenetic group of archaea [2]. Besides *N. equitans*, multiple other DPANN genera could be enriched or cultivated, and these diverse representatives all show an ectosymbiotic lifestyle [3–8]. The surge in metagenomic datasets has led to an expansion of DPANN diversity and revealed that relatively small genome size as well as reduced metabolic capabilities are common characteristics among these archaea [9, 10]. These findings suggest that the ectosymbiotic relationship with other archaea is a lifestyle that is shared among different—if not all DPANN lineages. Despite the apparent significance of the ectosymbiotic lifestyle for the evolution and ecology of archaea, the mechanistic aspects of DPANN-host interactions remain poorly understood. For instance, it is unclear how ectobiont-host interactions can be established in the presence of archaeal S-layer(s).

Here, we used complementary light and electron microscopy imaging techniques to characterize the ectosymbiosis between the recently described DPANN strain *Nanobdella aerobiophila* and its host [11] on a single-cell level. We would like to highlight a recent preprint reporting a complementary dataset [12].

## Materials and methods

### Strain and growth medium

*N. aerobiophila* MJ1^T^ (hereafter referred to as *Na*) was obtained from Deutsche Sammlung von Mikroorganismen und Zellkulturen as live co-culture with *Metallosphaera sedula* MJ1HA. The culture was incubated at 65°C. Medium for the culture contains (pH 2.5): 1.3 g l^−1^ (NH4)_2_SO_4_, 0.28 g l^−1^ KH_2_PO_4_, 0.25 g l^−1^ MgSO_4_·7H_2_O, 0.07 g l^−1^ CaCl_2_·2H_2_O, 0.5 g l^−1^ Yeast extract, 10 ml l^−1^ Allen’s trace element solution (composition from dsmz.de), 10 ml l^−1^ BME vitamin mixture (B6891, Sigma-Aldrich). Final concentration of trace elements in the medium: 1.8 mg l^−1^ MnCl_2_·4H_2_O, 4.5 mg l^−1^ Na_2_B_4_O_7_·10H_2_O, 0.22 mg l^−1^ ZnSO_4_·7H_2_O, 0.05 mg l^−1^ CuCl_2_·2H_2_O, 0.03 mg l^−1^ Na_2_MoO_4_·2H_2_O, 0.03 mg l^−1^ VOSO_4_·nH_2_O, 0.01 mg l^−1^ CoSO_4_·7H_2_O. BME vitamin mixture was not added to the medium for the time-lapse experiments.

The co-culture of *N. aerobiophila* MJ1^T^ and its host, *M. sedula* MJ1HA grew as white sediment developing on the bottom of a tube during 4 days of incubation.

### Time-lapse light microscopy

*Na*-host co-cultures were imaged using a Smart substrate (SmS-R-4) chamber heated by the VAHEAT system (Interherence) to 65°C on a Zeiss Observer Z1 microscope equipped with a Plan-Apochromat 100x 1.40 oil objective. Movies of 5 min were recorded at a rate of 1 frame per 0.6 s.

For image analysis, the grayscale of the movies was inverted and selected *Na* cells were subsequently automatically tracked using the plugin TrackMate 7 [13]. Tracks of low quality and those of *M. sedula* cells were discarded manually or by adapting the detection threshold. Swimming/non swimming cells were counted manually using the Fiji cell counter.

### Plunge freezing and cryo-ET data collection

Prior to freezing, an aliquot of the cells was collected and transferred from the culturing tube to a pre-warmed 1.5 ml microcentrifuge tube using a pre-warmed serological pipette. Until vitrification, the aliquot was kept at 65°C. Immediately before freezing, the cell suspension was mixed (10:1 v/v) with fiducial markers (Protein A - 10 nm Gold Conjugate, Cytodiagnostics, Canada) in fresh microcentrifuge tube. A total of 3.5 μl of the mixture were applied to glow-discharged EM copper grids (R 2/1, Quantifoil) for automated backside blotting at 22°C for 5 s and plunged into liquid ethane/propane (37%/63%) using a Vitrobot Mark IV (Thermo Fisher Scientific). The frozen grids were stored in liquid nitrogen.

CryoET data were collected using a Titan Krios G4 (Thermo Fisher Scientific) cryo-electron microscope with an accelerating voltage of 300 kV, equipped with a BioContinuum imaging filter and a K3 direct electron detector (Gatan). Tilt series were acquired using SerialEM with a dose-symmetric tilt scheme covering a range from −60° to +60° (2° increments). The defocus was −8 μm and a range (from −3 to −7 μm with 0.5 μm increments) for 54 and 42 other tomograms, respectively. The total electron dose was 171 and 129 e^−^/Å^2^ for 54 and 42 other tomograms, respectively.

### Data processing

The tilt series were drift-corrected using the alignframes command in IMOD [14]. Corrected tilt series were used to reconstruct 4x binned cryo-tomograms using IMOD with the back projection method. Reconstructed tomograms were CTF-deconvolved and filtered using IsoNet to achieve better contrast for visualization purposes [15]. The filtered tomograms were segmented using Dragonfly according to the published protocol [16]. Results of segmentation were visualized using UCSF ChimeraX [17].

## Results and discussion

**Figure 1:**
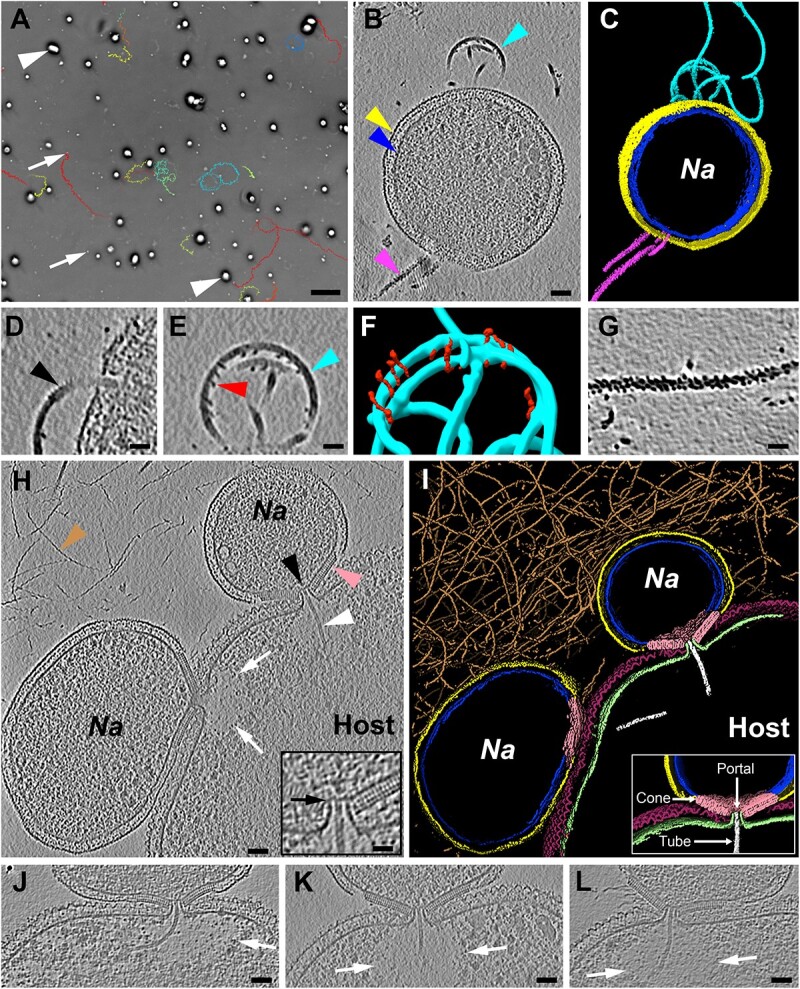
***Nanobdella aerobiophila* exhibits distinct life cycle stages.** (**A**) Time-lapse LM imaging of a co-culture of *Na* (examples indicated by arrows) with its host (*M. sedula*; examples indicated by arrowheads) reveals motility of non-attached *Na* cells. Shown is the last frame of a movie (5 min duration). Colored lines indicate the swimming paths of selected motile *Na* cells. See also Supplementary Movie S1. (**B/C**) CryoET imaging of free *Na* cells reveals S-layer (yellow arrowhead), cytoplasmic membrane (blue arrowhead), putative archaella (cyan arrowhead), and other extracellular appendages (magenta arrowhead). Shown is a slice (10.7 nm thickness) through a cryotomogram (B) and the corresponding model (C). Cyan, putative archaella; magenta, second type of extracellular appendage; yellow, S-layer; blue, cytoplasmic membrane. (**D–F**) Putative archaella consist of a short proximal corrugated segment (black arrowhead) and a long smooth distal segment (cyan arrowhead) decorated with thorns (red arrowhead). Shown are slices (5.3 nm thickness) through cryotomograms (D/E) and a model (F). Cyan, putative archaella; red, thorn-like features. (**G**) The second distinct type of extracellular appendage exhibited a rough surface. Shown is a slice (5.3 nm thickness) through a cryotomogram. (**H/I**) *Na* cells interacting with a host reveal an elaborate attachment organelle formed between ectobiont (*Na*) and host (*M. sedula*). Shown is a slice (10.7 nm thickness) through a cryotomogram (H; the inset shows a magnified view) and the corresponding model (I). The attachment organelle is formed by a repetitive cone-like structure (pink arrowhead), an intercellular portal (black arrowhead), a barrier (black arrow), and a tube (white arrowhead). Inset in (I) shows main modules of the attachment organelle). White arrows point to the depleted cytoplasm in the host close to the attachment site. Brown arrowhead points to the abundant thread-like extracellular appendages that are most likely associated with the host. Brown, abundant thread-like extracellular appendages; yellow, *Na* S-layer; blue, *Na* cytoplasmic membrane; pink, cone-like structure; white, tube; purple, host S-layer; green, host cytoplasmic membrane. (**J–L**) Shown are more examples of the attachment organelles with the conserved overall architecture and the flexibility of the tube curvature. White arrows point to an area with depleted host cytoplasm. Thickness of the slices is 10.7 nm. Scale bars: A, 10 μm; B–C/H/K–M, 50 nm; D–G, inset in H, 25 nm.

*Na* cells have been described as small cocci (0.2–0.5 μm in diameter) growing as obligate ectosymbionts with their much larger (0.8–1.6 μm coccus) host *M. sedula* at 65°C [11]. To characterize this ectosymbiotic relationship in more detail, we propagated a co-culture and imaged the cells at an early exponential time-point by time-lapse light microscopy (LM) on a temperature-controlled stage. We found that ~35% of the ectobionts in our analyzed movies were attached to host cells. In contrast to previous reports [11, 18], a 26% fraction of non-attached *Na* cells (n = 363) exhibited motility (Fig. 1A, Supplementary Movie S1). Despite their relatively slow speed of 0.37 ± 0.14 μm/s, individual *Na* cells were able to follow individual swimming trajectories of 9.05 ± 5.93 μm within a 5 min time frame. While archaella and other cellular appendages have been detected for *Na* and other DPANN previously, motility of unattached cells has not been reported for any DPANN strain so far. We speculate that motility in free *Na* cells increases the chance of encounters with host cells.

To reveal potential developmental adaptations of free and attached *Na* cells, we characterized the cellular architecture in a frozen-hydrated near-native state by cryo-electron tomography (cryoET) imaging [19, 20]. We plunge-froze a co-culture on EM grids and recorded cryotomograms of *Na* cells that were free/non-attached (n = 7) and attached to host cells (n = 109), respectively. Free *Na* cells appeared as relatively small cells with a diameter of 459 ± 64 nm (n = 7) and a typical archaeal cell envelope with a cytoplasmic membrane and an S-layer (Fig. 1B/C), consistent with previous observations [11, 18]. The cells frequently possessed two distinct types of filamentous extracellular appendages (Fig. 1B/C). The diameter (9 ± 1 nm, n = 58) of one type of appendage (seen in 57% of the free cells; n = 7) exhibited general similarity to archaella [21, 22]. This is consistent with the presence of archaellum genes in the genome and the detection of the major archaellin as the second most highly transcribed gene [18]. The putative archaella were composed of a proximal segment (76 ± 10 nm length, n = 13) that resembled a corrugated tube (Fig. 1D), and a long distal smooth segment that was sporadically decorated with thorn-like densities (Fig. 1E, F), features that have not been reported for archaella from other organisms. The surface of the second type of appendage (seen in 29% of free cells, n = 7) appeared rougher, and its identity is unknown (Fig. 1G). The diameter of the second type of appendage was 15 ± 3 nm (n = 15).

When imaging host-attached *Na* cells, we observed up to four ectobionts attached to an individual host cell. The general *Na* cellular architecture (476 ± 79 nm cell diameter, n = 109) was similar to the free cells with cytoplasmic membrane and S-layer. The putative archaellum and the second type of appendage were also found but only in 14 and 5% of ectobiotic cells (n = 109), respectively. However, we found one major distinguishing cellular feature: Each *Na*-host attachment site revealed an intricate interface complex (n = 109), which we refer to as “attachment organelle.” Several structural modules seemingly integrate the cellular components of *Na* with those of the host. The most prominent structural module is a cone-like structure with an opening (22 ± 1 nm, n = 89) at the vertex (Fig. 1H–L). This cone module is composed of repetitive densities and appears to be located on the outer surface of the *Na* cytoplasmic membrane, replacing the absent S-layer in this region of the cell envelope. The cone is also in close proximity to the S-layer of the host and may interact with it. The second structural module is a “intercellular portal,” which appears as an outward protrusion of the host cytoplasmic membrane that is inserted into the opening of the cone. The continuity of the cytoplasms of *Na* and host is interrupted by a barrier-like structure (Fig. 1H). Finally, 81% of all observed attachment organelles (n = 109) exhibit a further structural module, namely a thin tube (7 ± 1 nm, n = 80), which is often bent and extends from the barrier in the intercellular portal into the cytoplasm of the host (up to 360 nm in length). In 68% of the attached host cells (n = 109), the density of the cytoplasm seemed depleted of macromolecular complexes in the region close to the attachment site (Fig. 1H–L, Supplementary Data Fig. S1). The tube and the low-density host cytoplasmic volume were previously observed in electron microphotographs of the *Na*-host cellular associations [11]. Such multi-modular organization of intercellular contact was not found in other DPANN-host associations in previously published cryoET studies [23–25].

In conclusion, our data suggest the existence of an ectobiotic life cycle with two distinct stages. First, the host-seeking stage features unattached *Na* cells that possess archaella and exhibit swimming motility, a characteristic that has not been reported for any DPANN archaeon to date. This is intriguing, as the assembly and rotation of an archaellum are ATP-dependent [26, 27]. Due to their reduced genome, however, *Na* can likely not produce ATP at a similar rate as the host, because the genes of the ATP synthase could not be identified in its genome [11, 18]. Therefore, one could envision a scenario in which the *Na* cells assemble an archaellum while being attached to the “old” host and accumulate ATP to then be able to swim and find a new host before their ATP pool is depleted. An alternative scenario could involve ATP production through substrate-level phosphorylation, because the *Na* genome encodes genes of the Embden–Meyerhof–Parnas pathway, which were found to be expressed except genes for conversion of glucose to glucose 6-phosphate [11, 18]. Motility may increase the chance of encountering a host in the environment and may help to reduce the time spent in the free-swimming state as much as possible.

In the second stage, *Na*-host interactions are mediated by the remodeling of the *Na* and host cell envelopes and the formation of an elaborate attachment organelle. The architecture of the attachment organelle and the absence of immediate death of the host suggest a rather long-lived interaction/association. The attachment organelle likely evolved a mechanism to control transport between both cells instead of generating a conduit for a fused cytoplasm. We speculate that the attachment organelle (possibly via the tube) in fact allows for the directed transport of ectobiont enzymes into the host and/or the passage of metabolites such as amino acids, sugars, nucleotides, co-factors and vitamins from the host to the ectobiont. This is consistent with the difference in cytoplasmic density seen in the host close to the interaction site.

Reduced genomes, the ectobiotic lifestyle, and the intricate ectobiont-host interactions are thought to be trademark characteristics of all DPANN archaea. Our data will serve as a framework for future studies to determine the molecular identity of the attachment organelle and elucidate its evolutionary origin in ectobiotic archaea.

## Supplementary Material

Supplementary_Movie_S1_wrae076

Supplementary_Data_Figure1_wrae076

## Data Availability

Example tomograms (EMD-19886 to EMD-19893) were uploaded to the Electron Microscopy Data Bank.
