# Supplementary material for: Distinct life cycle stages of an ectosymbiotic DPANN archaeon
Source: ISME J. 2024 May 1;18(1):wrae076. doi: 10.1093/ismejo/wrae076 (PMC11104419; doi:10.1093/ismejo/wrae076)
Supplement: Supplementary_Data_Figure1_wrae076 [file supplementary_data_figure1_wrae076.pdf]

Supplementary Data

Distinct life cycle stages of an ectosymbiotic DPANN archaeon

[1#]Vasil A. Gaisin, [2]Marleen van Wolferen, [2] Sonja-Verena Albers, [1#]Martin Pilhofer

[1]Department of Biology, Institute of Molecular Biology & Biophysics, Eidgenössische Technische Hochschule Zürich, Otto-Stern-Weg 5, 8093 Zürich, Switzerland

[2]Molecular Biology of Archaea. Institute of Biology, University of Freiburg, Schänzlestr. 1, 79104 Freiburg, Germany

#: corresponding: pilhofer@biol.ethz.ch, vasil.gaisin@mol.biol.ethz.ch

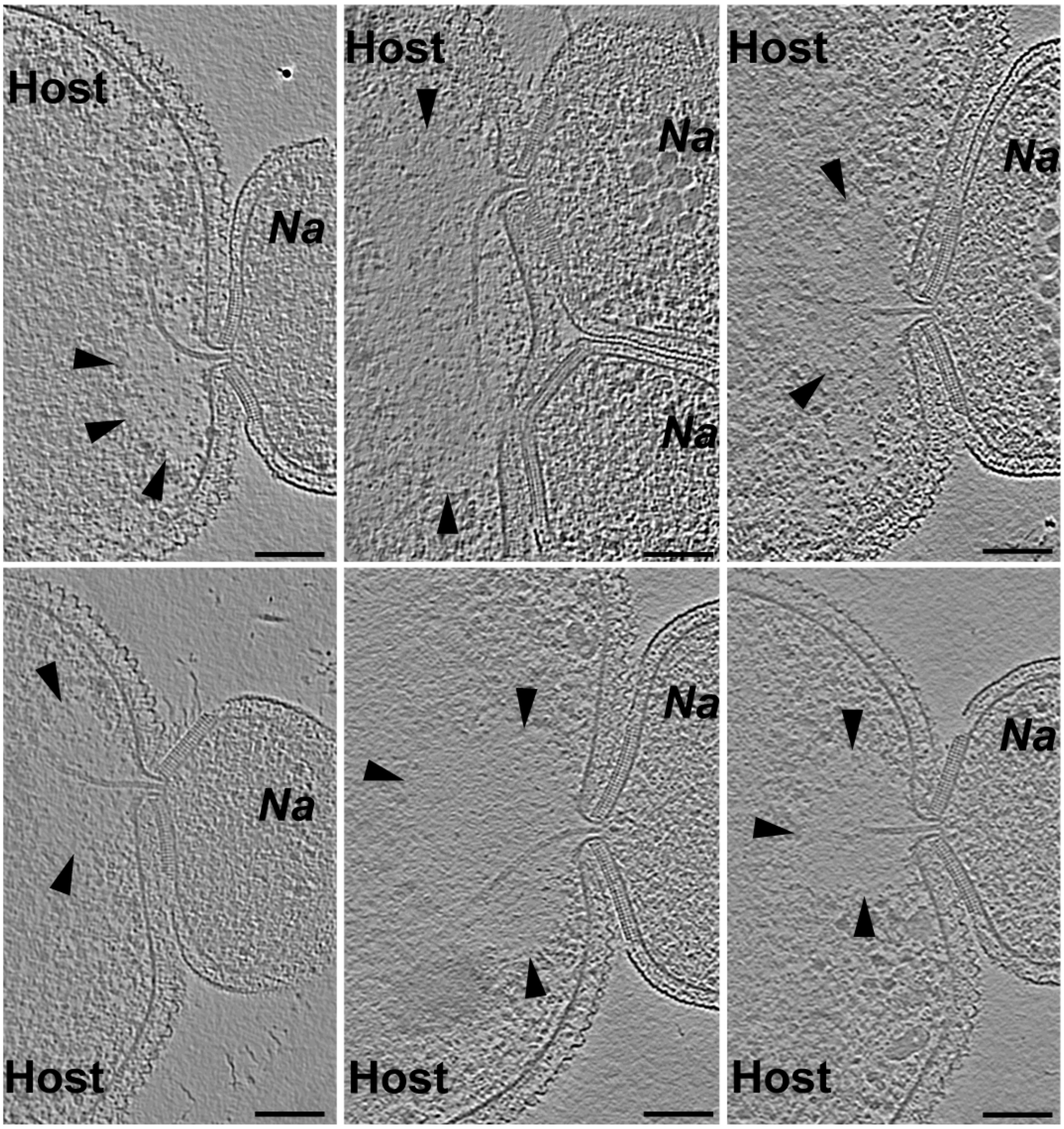

Supplementary Data Figure 1. Shown are more examples of the attachment organelles and depleted host cytoplasm. Slice through cryo-electron tomograms of attached cells of *N. aerobiophila* (arrowheads point to depleted host cytoplasm): Na, *N. aerobiophila* cell; host, *M. sedula* cell. Scale bars, 100 nm.

Supplementary Movie S1. Time-lapse light microscopy imaging.

Shown is a time-lapse movie recorded on a 65 °C heated stage (5 min duration, 1 frame per 0.6 s). Non-attached/free *Na* cells show motility. Colored lines indicate the tracks of selected motile *Na* cells.
